# Obituary—Dr. J. L. Nourse

**Published:** 1866-09

**Authors:** 


### OBITUARY.

We have just received the painful intelligence of the death of Dr. J. L. Nourse, of Cloverport, Ky. Of the particulars we have learned nothing, except that his sickness was not of long duration.

This is a loss that will be felt by the whole profession, but especially by that part of it in that vicinity. Dr. N. was one of the growing men in the profession. He was at the meeting at Boston, in apparently as good health and spirits as any one.

We suppose a tribute to his memory will be made by some one who had been longer acquainted with him than we.

